# Transcriptomic Analysis Brings New Insight into the Biological Role of the Prion Protein during Mouse Embryogenesis

**DOI:** 10.1371/journal.pone.0023253

**Published:** 2011-08-15

**Authors:** Manal Khalifé, Rachel Young, Bruno Passet, Sophie Halliez, Marthe Vilotte, Florence Jaffrezic, Sylvain Marthey, Vincent Béringue, Daniel Vaiman, Fabienne Le Provost, Hubert Laude, Jean-Luc Vilotte

**Affiliations:** 1 INRA, UMR1313, Génétique Animale et Biologie Intégrative, INRA, Jouy-en-Josas, France; 2 INRA, UR892, Virologie et Immunologie Moléculaires, INRA, Jouy-en-Josas, France; 3 INSERM, U567, Institut Cochin, Paris, France; Thomas Jefferson University, United States of America

## Abstract

The biological function of the Prion protein remains largely unknown but recent data revealed its implication in early zebrafish and mammalian embryogenesis. To gain further insight into its biological function, comparative transcriptomic analysis between FVB/N and FVB/N *Prnp* knockout mice was performed at early embryonic stages. RNAseq analysis revealed the differential expression of 73 and 263 genes at E6.5 and E7.5, respectively. The related metabolic pathways identified in this analysis partially overlap with those described in PrP1 and PrP2 knockdown zebrafish embryos and prion-infected mammalian brains and emphasize a potentially important role for the PrP family genes in early developmental processes.

## Introduction

The Prion protein, PrP, has been the focus of intensive research for decades due to its pivotal role in transmissible spongiform encephalopathies, a group of infectious neurodegenerative diseases of animal and human characterized by the accumulation of a pathological form of the protein (PrP^Sc^) [1]–[3]. The physiological function of this ubiquitously expressed protein is still unclear. Various roles in neuroprotection, cellular homeostasis, response to oxidative stress, cell proliferation and differentiation, synaptic function and signal transduction have been proposed [4]–[7]. Even the sub-cellular localization of this glycosyl-phosphatidyl-inositol-anchored cell surface glycoprotein remains a subject of debate leading to yet other purported physiological processes involving PrP ([8] for exemple). The difficulty to define a role for this protein partially comes from the observation that *Prnp*-knockout mice [9], [10], cattle [11] and goat [12] suffer from no drastic developmental phenotype. Similarly, invalidation of this gene in adult mouse neurons does not affect the overall health of the mice [13], [14]. It has been hypothesized that another host-encoded protein is able to compensate for the lack of PrP [15] or, if not redundant, that PrP has no physiological function [16].

Comparative transcriptomic and proteomic analyses of adult brain from *Prnp*-knockout mice did not reveal drastic alterations if any [17], [18], supporting the above-mentioned hypothesis. A recent transcriptomic study performed on the hippocampus of wild-type and *Prnp* ^−/−^ new born (4–5 day-old) and adult (3 month-old) mice, revealed only a moderate alteration of the gene expression profile [19]. On the other hand, developmental regulation of the mouse *Prnp* gene suggested possible involvement of PrP in embryogenesis [20]–[23]. Its implication in hematopoietic [24], [25], mesenchymal [26], neural [27], cardiomyogenic [28] and embryonic [29], [30] stem cell proliferation, self-renewal and differentiation was also recently highlighted.

In zebrafish, the Prnp gene is duplicated and encodes proteins PrP1 or PrP2. PrP1 or PrP2 loss-of-function were found to be detrimental to zebrafish embryogenesis and survival [31]–[33]. Furthermore we found in a previous study that PrP and its paralog Shadoo are required for early mouse embryogenesis as embryonic lethality was observed at E10.5 in *Sprn*-knockdown, *Prnp*-knockout embryos [34].

Altogether, these data suggest that even though PrP knockout is not lethal, the physiological role of PrP may have to be investigated at early developmental stages rather than in adults, or in specific cell types such as adult stem cells. The aim of this study is to assess the potential transcriptomic incidence of *Prnp*-gene invalidation at early embryonic stages (6.5 and 7.5 dpc.).

## Results and Discussion

### *Prnp*-invalidation induces transcriptomic alterations at E6.5 and E7.5

Pools of FVB/N and FVB/N *Prnp*-knockout embryos were collected at E6.5 and E7.5 and their RNAs analyzed by RNAseq. These two developmental time-points were chosen according to the previously observed lethality in FVB/N *Prnp*-knockout, *Sprn*-knockdown embryos occurring before E10.5 [34] that was already substantial at E8.5, the gastrulation stage in mouse (BP and MV unpublished data). Seventy-three and 263 differentially expressed genes were detected between the two genotypes studied at E6.5 and E7.5, respectively (Table 1 and S2), representing 0.23 and 0.78% of total expressed genes. The majority of differentially regulated genes were under-expressed in *Prnp*-knockout versus wild-type embryos, 71.2 and 89.7% at E6.5 and E7.5, respectively (Table 1).

**Table 1:** Number of differentially expressed genes in Prnp-invalidated mouse embryos.

	E6.5	E7.5
**Total number of Differentially expressed genes**	**73**	**263**
**Up-regulated**	**21 (28,76%)**	**27 (10.2%)**
>10 fold	**4 (5.47% of deregulated genes, 19.04% of up-regulated)** LOC676933, Pax8, Mgat4c, Ptrf	**7 (2.66% of deregulated genes, 25.92% of up-regulated)** Serpina1e, Napsa, Prss28, Prss29, NM_024283.2, Slco1a6, Prap1
**Down-regulated**	**52 (71.23%)**	**236 (89.7%)**
>10 fold	**29 (39.72% of deregulated genes, 55.76% of down-regulated)** Speer2, Fam186b, Rax, H2-M10.1, LOC633979, Magel2, LOC100046045, Cdh22, Myoz1, LOC100043825, Rmrp, Indol1, Scrt2, NM_175674.2, Dnajb5, Pcdh19, Mib2, LOC100046008, Scn3b, Rufy3, Stac3, Fam154b, EG666182, LOC633979, Hist4h4, Chsy3, XM_130735.7, LOC435145, Hmx2	**47 (17.87% of deregulated genes, 19.91% of down-regulated)** NM_177599.3, Msln, Hspb7, Kcnd3, Slpi, Tnfaip3, SecTm1b, Cst9, XM_001478533.1, Samd12, XR_032778.1, Corin, Havcr2, Ppp1r3c, Cyp2j11, Lsamp, Hsd11b1, Slc6a12, Lyve1, Ugt1a1, Cldn1, Emcn, Gpr115, Klk14, XR_034037.1, Anxa8, Psca, Lims2, Ly6Cc1, Ugt1a6b, Ly6c2, misc, Ly-6c, Pdzk1ip1, Tmem154, Ly6A, Tnfrsf11b, Gpr64, Lbp,Gpr115, Pglyrp1, Angpt4, Des, Tdo2, NM_172777.2, Dmkn, Alox5,

Genes deregulated more than 10 fold are listed.

To be pointed out, the *Prnp* mRNA itself was not significantly differentially expressed at either E6.5 or E7.5. This observation is explained by the fact that the knocking out was performed by insertion of a neomycin-resistance gene within exon 3 of the *Prnp* locus [9]. The *Prnp* gene remains transcribed, although at an approximately 2-fold lower level as observed by Northern blotting and according to the RNAseq data (data not shown), but the resulting mRNA no longer encodes for PrP [9].

**Figure 1:**
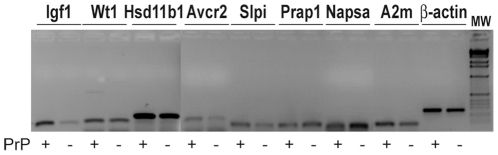
PCR analysis of differentially expressed genes. Gel electrophoresis showing the amplification signals obtained by RT-PCR for 8 genes identified by RNAseq as being differentially expressed. The RT-PCR analyses of these 8 genes were congruent with the results obtained by RNAseq. Beta-actin was used as an internal control. PrP + and −: pools of E7.5 FVB/N and FVB.N *Prnp*-knockout embryo RNAs, respectively.

Ten genes were arbitrarily chosen for confirmation by RT-PCR. This was performed on E7.5 RNA samples different from those used for RNAseq experiments. The amplification signals obtained for the Ptrf and Prss28 transcripts were too low to be analyzed (data not shown). The RT-PCR analyses of the remaining 8 genes were congruent with the results obtained by RNAseq (Figure 1 and Table S2), confirming that comparative RNAseq analysis is a quantitative approach [35].

Twelve genes were differentially expressed at both embryonic stages of which 5 were consistently over-expressed in *Prnp*-knockout embryos (Table 2). Of note, these 5 genes, Prss28, Prss29, Napsa, MmP7 and XM_001477507.1, a transcript similar to that of ISP-2, share proteolysis activities and can modulate cellular adhesion and extracellular matrix deposition [36].

**Table 2:** Genes differentially expressed at both E7.5 and E6.5.

Name	E7.5 (WT versus KO)	E6.5 (WT versus KO)
Ptrf	0.120	13.726
Rmrp	4.18	9.153
Napsa	26.437	9.153
4933425M15	6.025	0.062
Prss28	16.875	5.26
Prss29	16.687	9.117
Mib2	5.011	0.067
LOC100047285	9	9.477
Acvr1c	3.401	0.181
Mmp7	7.781	6.44
Rpph1	3.087	0.114
LOC677333	2.452	0.186

Numbers refer to fold change.

*In silico* analysis of the pathways affected by the observed transcriptomic alterations was undertaken to gain new insight into the biological function of PrP during mouse embryogenesis.

### Convergence of *Prnp*-invalidation induced transcriptomic alterations between mouse and Danio rerio embryos

**Figure 2:**
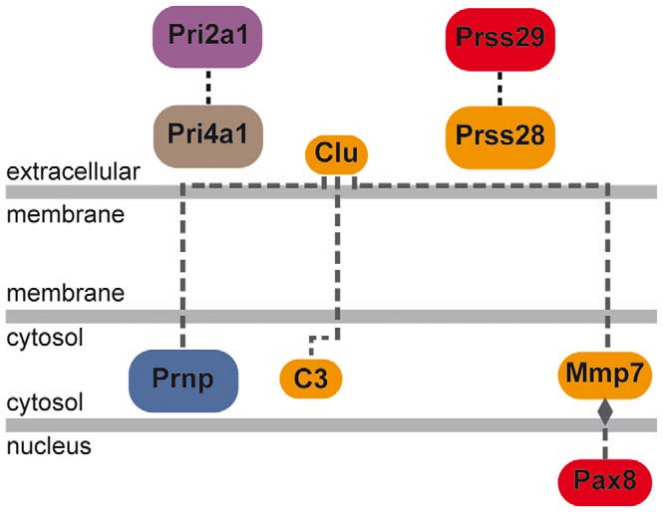
Differentially expressed gene pathway at E6.5 linking Pax8 and Mmp7. A pathway was generated by the GEPS program from Genomatix that connects differentially expressed genes found at E6.5. It suggests a modulation of the matrix metalloprotease Mmp7 by PAX8. (See Figure S2 for detailed Genomatix network legend).

**Figure 3:**
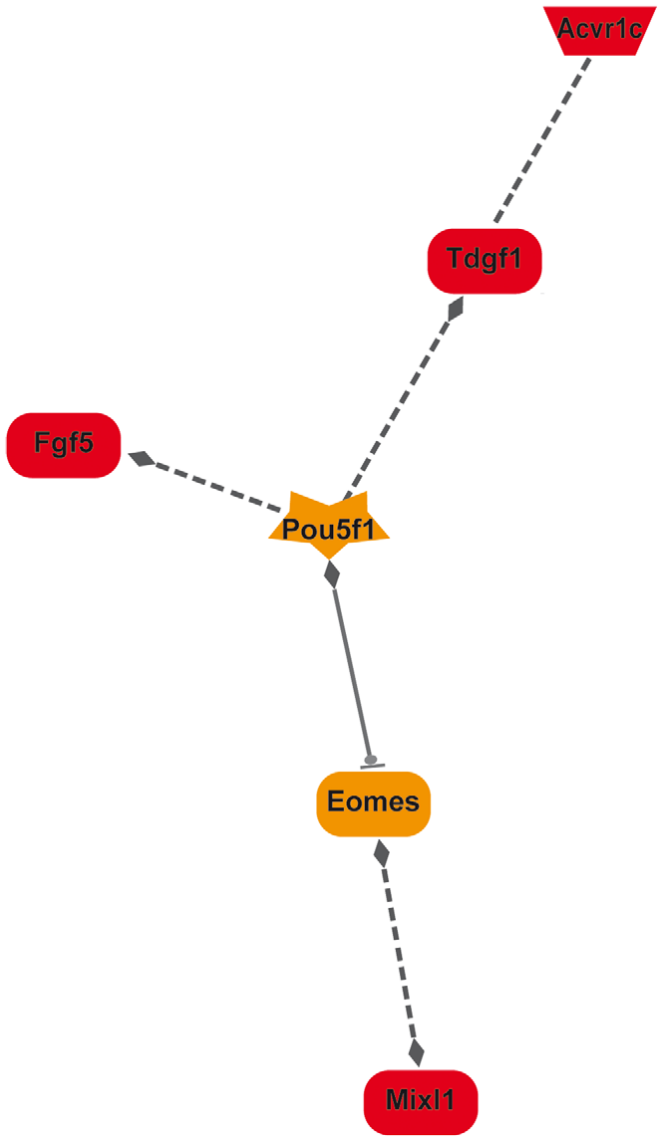
Pou5f1 could modulate expression of other observed key factors at E7.5. A GEPS network connecting the up-regulated genes at E7.5 was detected. It shows a potential regulation by Pou5f1 of the over-expressed key regulatory factors. (see Figure S2 for detailed genomatix network legend).

As mentioned above, 5 of the 12 genes that were differentially expressed at E6.5 and E7.5 were over-expressed in *Prnp*-knockout embryos and encode endopeptidases (Tables 2, 3 and S2). Mmp7 deregulation could be a consequence of the up-regulation of the Pax8 transcription factor at E6.5, as suggested by GEPS analysis (Figure 2), although to our knowledge a direct functional link between these two factors remains to be demonstrated. Endopeptidases can modulate the biological levels of cadherins and catenins and it was hypothesized that such induced alterations in cell-cell communication were at the origin of the arrested gastrulation observed in PrP1-depleted zebrafish embryos [31]. At E6.5, cadherin 22 and protocadherin 19 were indeed down-regulated in *Prnp*-knockout mouse embryos, suggesting an induced perturbation of cell movement [37], as described in zebrafish. Moreover, a correlation between lack of PrP and down-regulation of cadherins was recently described in the mouse hippocampus [19]. Modified cellular adhesion and cell proliferation pathways were detected at E7.5 (Table 3) [36], [38]. These networks highlight potential key regulatory roles of the growth factor Fgf5, Igf1 and Tdgf1 proteins, expression of which was significantly modified in the absence of PrP (Supplementary data Table S2 and data not shown). Biological links between some of these proteins and PrPc have already being described in adult tissues [39]–[41]. However, their modified expression could be indirectly linked to that of PrP, and induced by overexpression of Pou5f1 (Figure 3). This transcription factor is up-regulated in *Prnp*-knockout E7.5 embryos (Table S2) and has been associated with *FGF5* and *TDFG1* gene regulation [42], [43]. Expression of Pou5f1 and PrP was recently found to be correlated in differentiating mouse ES cells [30]. The Igf1 deregulation might be a consequence of the observed deregulation of Wt1 ([44] and Table S2), although the link between the Wt1 transcription factor and Igf1 regulation remains hypothetical. On the other hand, the up-regulation of Fgf5 could have in turn down-regulated that of Igf1 in the absence of PrP, as already observed [39]. Thus, the *Prnp*-knockout induced deregulation of Pou5f1 might be the trouble spot at the origin of these networks (Figure 3).

**Table 3:** Summary of the highest represented functional groups of genes from the differentially expressed gene list at E7.5.

Category	Genes	Nb of genes
Proteolysis	**Prss28,Prss29,Napsa,Mmp7,XM_001477507.1(similar to ISP-2)**,Ctsk,Ctso	7
Protease inhibition	Slpi,Cst9,A2m,**Serpina1e**	4
Biological adhesion	Igfbp7,Angpt2,Smoc2,Epdr1,Sned1,Igsf11,Nrcam,Lsamp,Thy1,Adam12,Cd36, Cd34,Lamb2,Gpnmb,**Tdgf1**,Fbln2,Msln,Lyve1,Cldn1,Cldn5	20
Nervous system development	Igf1,**Fgf5**,Bdnf,**Apob**,Aqp1,Sgk1,Bmp2,Atoh8,Tgfbr2,Cldn5,Cldn1,Gja1,Thy1, **Eomes**,Ednrb,Mt3,Tacc1,Hoxd10,Hdac9,Nrcam,Corin,Mal,Lamb2	23
Apoptosis	Tnfrsf11b,Igf1,Bdnf,Ednrb,Bmp2,Irak3,Tlr4,Pglyrp1,Pmaip1,Casp12,Tnfaip3, Gja1,Sgk1,Wt1,Srgn,**Acvr1c**,Anxa1,Cryab,Cryaa,Mal	20
Cell proliferation	**Fgf5**,Igf1,Bdnf,Ednrb,Lgr4,Tgfbr2,Tlr4,Ptges,**Acvr1c**,Fosl2;Bmp2 Ptgs1,Gja1,**Mmp7**,Nampt,Sat1,Anxa1,Pmaip1,**Tdgf1**,Tacc1	20
Inflammatory and innate immune response	Tlr4,Serping1,Cd55,Bmp2,Tlr3,Lbp,Alox5,Ggt5,Anxa1,Hdac9,Cd36,Pglyrp1,Irak3	13
Heart formation and blood vessel development	**Apob**,Cd36,Igf1,Wt1,Angpt2,Tgfbr2,Gja1,**Tdgf1**,Thy1,Cdx4,Tnfaip2	11
Vascular diseases	Atp8b1,Ugt1a1,**Slco1a6**,Rgs5,Tnfaip2,Rgs2,Nrcam,Pmaip1,Lsamp,**Pou5f1**,**Mixl, Tdgf1**,Casp12,Lbp,Havcr2,Tlr4,Irak3,Tlr3,Fstl1,Add3,Ptn,**Fgf5**,**Penk**,Mt3,Bdnf, Tgfbr2,Fbln2,Fbn1,Mfap5,Col5a2,Dcn,**Lum**,Ramp3,Sparcl1,Timp3,Ednrb,Gpx3, Adam12,Abp1,Mgp,Bmp2,Fosl2,Vsig2,Cyp11b1,Igfbp7,Nampt,Sat1,Angpt2,Slpi, Igf1,Alox5,Anxa1,S100a4,Pglyrp1,Ptgs1,Ptges,Tnfaip3,Hdac9,Hsd11b1,Slc2a12,Srgn, **Apob**,Vldlr,Cd36,Tnfrsf11b,Ctsk,Cxcl14,Sgk1,Srd5a1,Bche,Gda,Abat,A2m,Gja1,Des,Dtna, Cryab,Cd34,Il6ra,Abcd1,Hoxa10,Sfrp4,Sfrp5,Dkk2,Wt1,Tacc1,Cldn1,Cldn10a,Cldn5, Lyve1,Emcn,Thy1,Cd55,Aqp1,Serping1,Ly6a,Ly6c1,Ly6c2,Gpihbp1	99
Response to oxidative stress	Gpx3,Tlr4,Ptgs1,Anxa1,Cryab,Cryaa	6
Matrix metalloproteinase	Angpt2,Slpi,Igf1,Timp3,Tlr4,Rgs2,Irak3,Ptges,Alox5,S100a4,Adam12,**Mmp7**, Aqp1,Nampt,Wt1,Hoxa11,Bmp2,Tgfbr2,Bdnf,Fosl2,Jam2,Cldn5,Tnfrsf11b	23
Prion disease	Bdnf,Igf1,Dcn,Bche,Tgfbr2,Srgn,Aqp1,Cd34,Thy1,Ly6a,Cryab,Anxa1,Ptgs1, Alox5,Tlr3,Tlr4,Casp12,Mt3	18

**UP-regulated genes are in bold type.**

At E7.5, the relative over-expression levels of Prss28, Prss29, Napsa, MmP7 and XM_001477507.1 in *Prnp*-knockout embryos were significantly elevated compared to those observed at E6.5 (Table 2 and S2). Protease inhibitors such as Slpi, Cst9, A2m were also down regulated at this stage, which could increase the activity of matrix metalloproteases [45], [46], while Serpina1e expression was highly elevated. However, Pax8 up-regulation was not in evidence at E7.5, neither were cadherin 22 and protocadherin 19 down-regulations, which might reflect an adaptation of the embryonic metabolism.

Overall, this transcriptomic analysis reveals a striking biological convergence between the PrP-knockout induced deregulation in early mouse embryos and that previously described in PrP1-invalidated zebrafish eggs [31]. The resulting outcome is not lethal in the mouse which, according to the results published by us [34], suggests a sufficient compensatory mechanism by the related Shadoo protein to the absence of PrP that sustains the embryonic development. So far, the developmental regulation of the *Sprn* gene and the biological properties of the protein have not yet been described in zebrafish. Such investigations could indirectly validate this hypothesis.

Invalidation of the later developmentally regulated PrP2-encoding zebrafish gene led to impaired brain and neuronal development [32]. Although it is reminiscent of the phenotype observed in the surviving mouse *Prnp*-knockout, *Sprn*-knockdown embryos [34], such phenotype has not been described for mammalian *Prnp*-knockout embryos. However, our transcriptomic analysis also highlights alterations of specific networks involved in nervous system development in the *Prnp*-knockout mouse embryos, as described below.

### The transcriptomic alteration in *Prnp*-invalidated mouse embryos evokes a negative image of that found in prion-diseased brains

Although prion-associated pathologies have been extensively studied, with detailed descriptions of the associated neuropathology, the underlying mechanism leading to neurodegeneration is still poorly understood ([47] for review). Among the existing debate is the question whether this pathology results from a PrP loss-of-function, a PrP^sc^ gain-of-function, a subversion of PrP function by PrP^SC^ or a combination of these three mechanisms [48]. The simple PrP loss-of-function hypothesis was not sustained by the observation of the limited and subtle phenotypes resulting from the gene invalidation in mammals [9]–[12].

Differentially expressed genes in prion-infected adult mouse brains have been identified by microarray analyses [49]–[51]. Although strain-specific responses were detected leading to some gene specificity, overall similar biological functions were highlighted in all experiments, such as cell growth and adhesion, proteolysis, protease inhibition, response to oxidative stress, inflammation, immune response, cell death and neurological disorders. In each of the identified pathways, genes that were also differentially expressed in our study were described. Indeed, similar biological networks were identified by Ingenuity and Genomatix analyses (Table 3). We have already mentioned specific pathways involving cell proliferation and adhesion as well as differentially expressed proteases and anti-proteases that distinguish *Prnp*-knockout embryos from their wild-type counterparts (Table 3). At E7.5, matrix metalloprotease, apoptosis, inflammatory response and response to oxidative stress networks were also revealed by *in silico* analyses. Two genes appear to be central to these networks, Igf1 and Tlr4 (Figure S1). As mentioned previously, deregulation of Igf1 has previously been observed in relation with PrP [39]. Invalidation of Tlr4 resulted in accelerated prion disease pathogenesis in transgenic mice [52], a result attributed to a modulation of the innate immune system response. Our data would suggest that PrP expression *per se* has a role in immune function in the absence of pathogenic prion.

**Figure 4:**
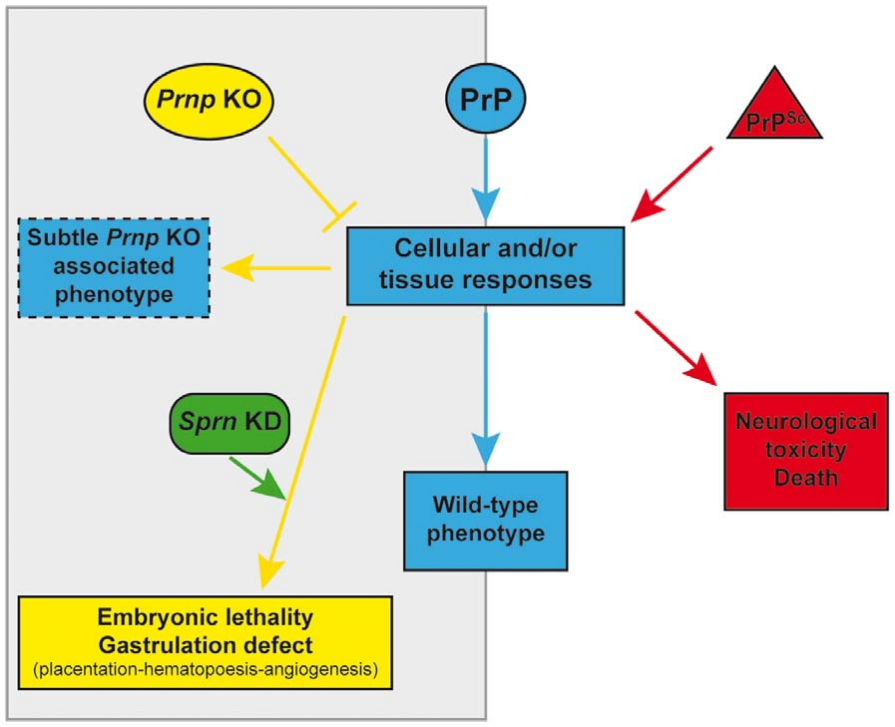
Schematic presentation of the mirror effect hypothesis. The activated cellular and/or tissue response in the presence of PrPSc (right side) is a negative image of that observed in the absence of PrP (left side). It suggests a mechanism of prion disease associated neurodegeneration in which the over activation or subversion (Harris and True, 2006) of PrP by PrPSc could lead to neurotoxicity. In Prnp knockout mice, the presence of the protein shadoo seems to be essential for an efficient compensatory mechanism and survival. In Prnp-knockout, Sprn-knockdown mice, an embryonic lethality was observed, this lethality could be caused by a gastrulation defect characterized by placentation and hematopoesis defaults.

Most of the differentially expressed genes were reported to be up-regulated in prion affected adult brains while they are down-regulated in *Prnp*-knockout embryos. For example, the up-regulation of Cathepsins, a family of lysosomal proteases, has been associated with neurodegenerative diseases, including Alzheimer's and prion diseases [49]. In contrast, our results demonstrate a down-regulation of Cathepsins in the absence of PrP. Overall; this observation suggests that the prion disease pathology does not mimic a PrP-lack of function. On the contrary, it seems that in the presence of infectious prions, the activated cellular or tissue response is a negative of that observed in the absence of PrP, suggesting that prions over-activate the normal PrP protein signaling, leading to neurotoxicity. This hypothesis may also relate to the observed down-regulation of the Shadoo protein in terminally prion-affected mouse brains. In zebrafish, this mirror effect would lead to embryonic lethality in the absence of either PrP1 or PrP2, while in mammals, a host-encoded protein, probably Shadoo [34], might allow for sufficient compensatory mechanisms to take place to sustain embryonic development (Figure 4).

### *Prnp*-invalidation induced transcriptomic alterations are consistent with a putative role of PrP during early embryogenesis

Invalidation of PrP induces transcriptomic alterations that can be related to several developmental processes. At E6.5, deregulation of transcription and chromatin-related biological process were detected, as exemplified by the down-regulation of various histone cluster genes in *Prnp*-knockout embryos (Table S2), possibly reflecting the above-mentioned perturbation of the cell proliferation process. It further highlighted the implication of PrP in the self-renewal and differentiation of stem cells [27], [29], [30]. Similarly, specific networks related to the development of the nervous system were detected (Table 3), further emphasizing the neuro-specificity of PrP signaling [7], as well as with other developmental processes such as odontoblastic/osteogenic and muscle development.

Our analysis pointed towards a major pathway involved in cardiovascular development, hematopoiesis and angiogenesis, with the identification of specific networks including vascular diseases, arteriosclerosis, blood vessel development and morphogenesis (Table 3 and data not shown). PrP has recently been shown to identify bipotential cardiomyogenic progenitors [28] and to be involved in the self-renewal of hematopoietic stem cells [24]. Its absence in *Prnp*-knockout embryos negatively affects the expression of Mesp1 (Supplementary data Table S2), a gene associated with the earliest signs of cardiovascular development [53], potentially capable of generating the multipotent cardiovascular progenitors and involved in the epithelial-mesenchymal transition [54]. *Prnp*-knockout embryos also had reduced expression of Timp-3, a gene that expands the multipotent hematopoietic progenitor pool [55], and that of Hoxa10, a gene also involved in this process [25]. *Prnp* invalidation increased the expression of the MixL1 transcription factor, capable of suppressing hematopoietic mesoderm formation and promoting endoderm formation [56], and that of TDGF1 that inhibits cell differentiation [57] (Figure 3). Finally, the absence of PrP also negatively affects the expression of various G protein-coupled receptors and doing so angiogenesis [58].

**Figure 5:**
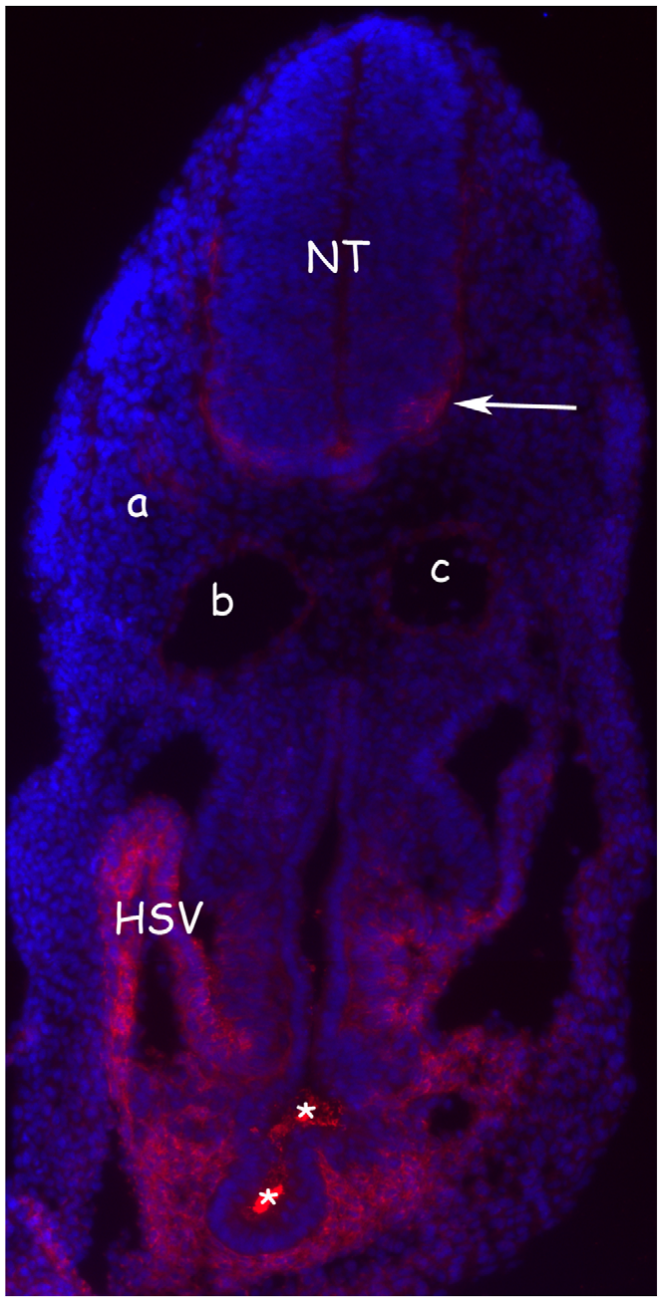
Expression of PrP in the embryo. At E9.5, PrP expression was observed in endothelial cells of blood vessels (a), including dorsal aortas (b, c) and in the developing heart with, as shown in this figure, a very intense signal observed in the sinus venosus. The saturated signal visible here (asterisk) is artefactual. HSV: horn of the sinus venosus. At E9.5, PrP expression in the nervous system is mainly detected in the lateral part of the neural tube (NT), the mantle zone (arrow): this zone is formed by cells undergoing differentiation that have migrated from the medial part of the neural tube where cells continue to divide (progressively during development, PrP staining in neural tube increases as the mantle zone thickened; our unpublished data).

These findings raise the question if and to what extent PrP is expressed in the cardiovascular system of the early embryo. Immunochemistry analysis could not be performed at E7.5 for technical reasons (see materials and methods). At E9.5, however, it revealed expression of PrP in the developing heart, as previously described using PrP-LacZ transgenic mice [22], with a particularly intense signal in the region of the sinus venosus, a part of the embryonic cardiovascular system draining the blood flow into the heart (the venous pole) (Figure 5). Interestingly, expression pattern of PrP in this region is reminiscent of that of *Islet1*, a transcription factor gene expressed by cardiogenic progenitors [59]. In addition, PrP expression was detected in the endothelium of blood vessels such as the dorsal aortas. Worthy of note, PrP expression in the cardiovascular system was of comparable intensity to that visible in the nervous system, especially the developing neural tube (Figure 5).

Expression of PrP in extra-embryonic tissue has been described and occurs at early developmental stages [22], [23], [60]. The transcriptomic perturbations observed in the *Prnp*-knockout embryos affect genes involved in placentation. Adam12, a candidate regulator in trophoblast fusion [61], is under-expressed as well as the Cysteine-Cathepsins which are essential for extra-embryonic development [62]. Activin receptors are also differentially expressed (Table S2). Activin promotes differentiation of mouse trophoblast stem cells [63] and its receptor controls trophoblastic cell proliferation [64]. Differential expression of other key regulators acting on placentation was observed such as that of fibroblast growth factors [65], brain-derived neurotropic factors [66] and proteins involved in the epithelial-mesenchymal transition, Pou5f1, TDGF1, Mesp1 [54], [67] and potentially Pax8 [68] (Table 3).

Overall, our analysis suggests that, through the deregulation of the above-mentioned transcription factors and key genes involved in the maintenance, renewal and differentiation of stem cells, PrP invalidation affects numerous developmental processes that take place around gastrulation. A biological effect of these deregulations is a perturbation of the cellular interaction and cell homeostasis. In zebrafish, it leads to a morbid phenotype. In mammals, compensatory mechanisms would reduce this phenotype and allow sustaining a nearly normal embryonic development. Recent data suggest that the prion-related Shadoo protein has a crucial role in this process in the absence of PrP [34]. The phenotype associated with the *Prnp*-knockout, *Sprn*-knockdown genotype was not clearly identified but our current data support the hypothesis of a lethal defect in early gastrulation in the absence of these prion-related proteins characterized by a default in placentation, angiogenesis and hematopoiesis (Figure 4). Current investigations are underway to assess this hypothesis.

## Materials and Methods

Animal experiments were carried out in strict accordance with the recommendations in the guidelines of the Code for Methods and Welfare Considerations in Behavioural Research with Animals (Directive 86/609EC). And all efforts were made to minimize suffering. Experiments were approved by the Committee on the Ethics of Animal Experiments of the author's institution, INRA (Permit Number RTA06-091). All animal manipulations were done according to the recommendations of the French Commission de Génie Génétique (Permit Number N°12931 (01.16.2003)). Total RNA was isolated from pools of whole FVB/N and FVB/N Prnp^−/−^ mouse embryos at stages E6.5 and E7.5 [9], [69]. RNA extractions were performed using the RNeasy Lipid Tissue Mini kit (Qiagen cat # 75842). RNA concentration was calculated by electro-spectrophotometry and the RNA integrity checked with the Agilent Bioanalyser (Waldbroom, Germany).

RNA samples of 5 microg, obtained from around 30 embryos each collected from 3 to 4 females, were sent to GATC Biotech SARL for RNAseq analysis. A standard cDNA library was derived from each sample, with colligation and nebulization of cDNA and adapter ligation. These cDNAs were analyzed on an Illumina Genome Analyzer II with raw data output of up to 350 Mb and 42,000,000 reads per sample and a read length of 36 bases (single read). Sequence cleaning was done using Seqclean (http://compbio.dfci.harvard.edu/tgi/sofware/seqclean_README). Cleaned reads were mapped to the NCBI mouse transcript database (ftp://ftp.ncbi.nih.gov/genomed/M_musculus/RNA/) using BWA software [70].

Differentially expressed genes between FVB/N and FVB/N *Prnp* ^−/−^ embryos were identified at 5% FDR using the DESeq software from the package R [71]. They were clustered using the software DAVID [72], [73], then classified in pathways by using Ingenuity (http://www.ingenuity.com/) and in networks and biological functions using the GEPS application of Genomatix (http://www.genomatix.de).

RT-PCR analyses were performed using 3 microg of purified RNA reverse transcribed with the SuperScript II Reverse Transcriptase kit (Invitrogen), according to the manufacturer's protocol. RT-PCR analyses were performed using a set of oligonucleotides located in different exons (Table S1) for each gene analyzed to avoid potential genomic DNA amplification. The PCR conditions comprised 35 cycles of 94°C for 30s, 60°C for 30 s and 72°C for 40 s. Amplified fragments were visualized under UV following size resolution by 1.5% agarose gel electrophoresis in the presence of ethidium bromide.

For immunohistochemistry, transverse sections of E9.5 FVB/N formalin-fixed embryos (14 microm-thick) were stained with Sha31 antibody [74]. Images were acquired using a Zeiss microscope. E9.5 FVB/N *Prnp* ^−/−^ embryos were used as negative controls. It should be noted that immunohistochemistry analysis performed on younger embryos led to an artefactual signal, thus preventing the study of expression of PrP at earlier stages.

## Supporting Information

Figure S1 **A network connecting the differentially expressed genes at E7.5.** A network connecting the differentially expressed genes from *Prnp* Knock-out embryos was identified by GEPS application from Genomatix in which Tlr4 and Igf1 occupy a central role alongside, but to a lesser extent, Cd34 and Thy1. Red color indicates up- and blue down-regulated genes, respectively (see supplementary data Figure 2 for detailed Genomatix network legend).
(TIF)

Figure S2 **Genomatix pathway system legend.** Description of the genomatix pathway system legend is given. It applies to the figures 2 and 3.
(TIF)

Table S1 **Primer sets used for PCRs.**
(DOCX)

Table S2 **List of differentially expressed genes in** ***Prnp*** **-knockout embryos.** Up-regulated genes are in green. Down-regulated genes are in red.
(DOCX)
