# Supplementary figures and images for: Transcriptomic Analysis Brings New Insight into the Biological Role of the Prion Protein during Mouse Embryogenesis
Source: PLoS One. 2011 Aug 15;6(8):e23253. doi: 10.1371/journal.pone.0023253 (PMC3156130; doi:10.1371/journal.pone.0023253)

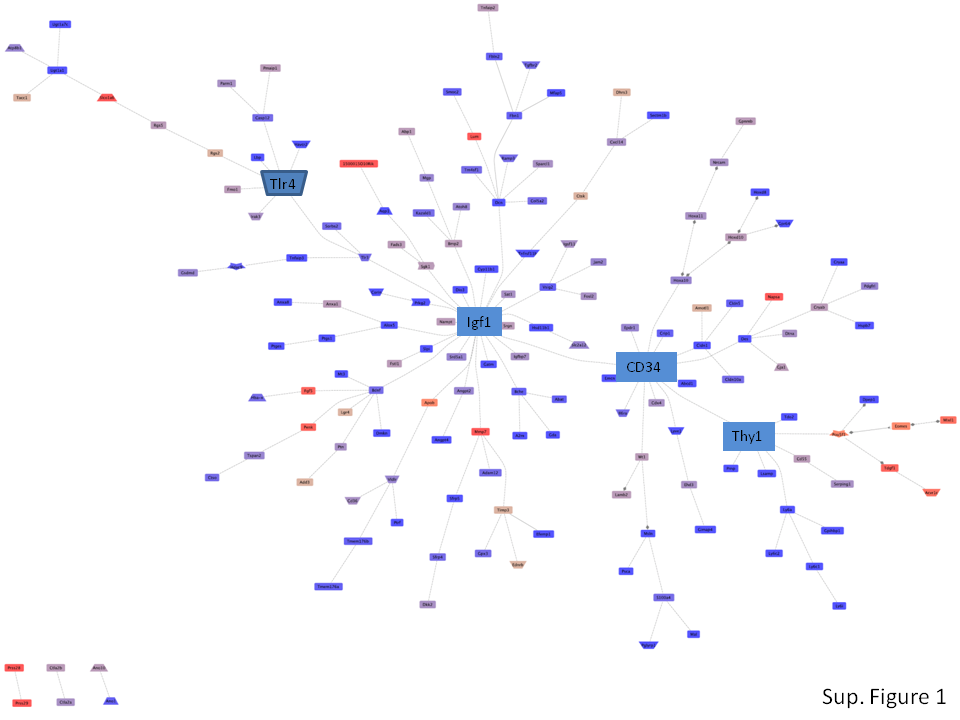

Supplement: Figure S1 — A network connecting the differentially expressed genes at E7.5. A network connecting the differentially expressed genes from Prnp Knock-out embryos was identified by GEPS application from Genomatix in which Tlr4 and Igf1 occupy a central role alongside, but to a lesser extent, Cd34 and Thy1. Red color indicates up- and blue down-regulated genes, respectively (see supplementary data Figure 2 for detailed Genomatix network legend). (TIF) [file pone.0023253.s001.tif]

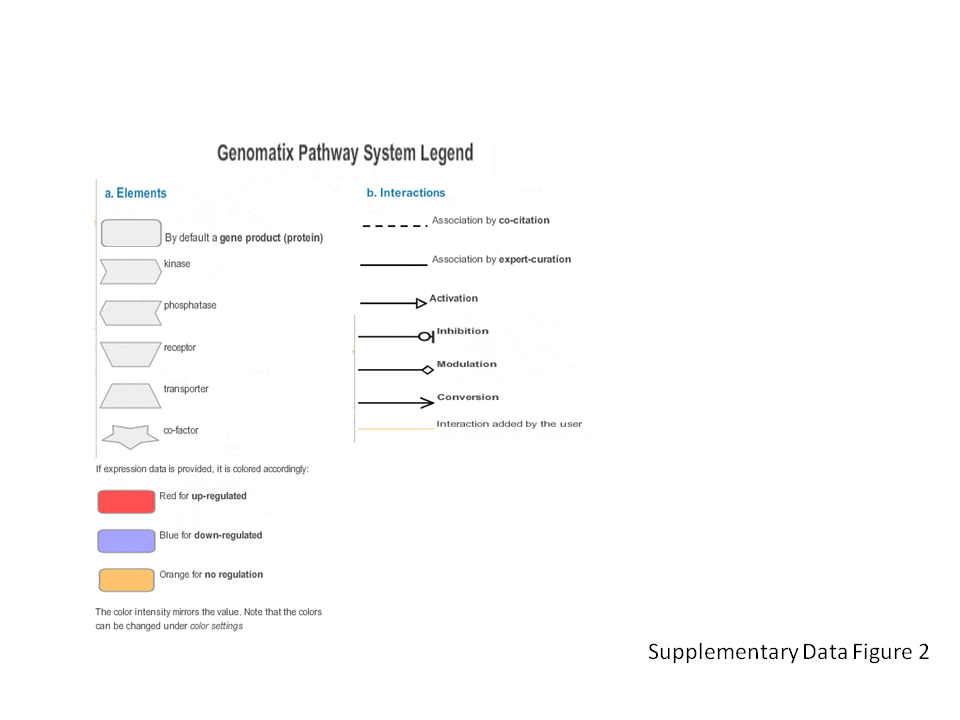

Supplement: Figure S2 — Genomatix pathway system legend. Description of the genomatix pathway system legend is given. It applies to the figures 2 and 3. (TIF) [file pone.0023253.s002.tif]
