# Supplementary material for: Transcriptomic Analysis Brings New Insight into the Biological Role of the Prion Protein during Mouse Embryogenesis
Source: PLoS One. 2011 Aug 15;6(8):e23253. doi: 10.1371/journal.pone.0023253 (PMC3156130; doi:10.1371/journal.pone.0023253)
Supplement: Table S1 — Primer sets used for PCRs. (DOCX) [file pone.0023253.s003.docx]

| Gene Name | Oligonucleotide sequences |
| --- | --- |
| Prss28 | CGACATGCTTTGTGCTGGCA |
|  | GCCCACCTGTATCCACTTGT |
| Napsa | TAACCTCACAGGCCAGGACT |
|  | GCTTGGGGATATCCAAGGCTT |
| Prap1 | CAAACAGAAGCCTGCAGCTG |
|  | GTTCTGGACCCTGAAGAGGA |
| Slpi | AATACAAGTGCTGTGAGGGTAT |
|  | AGAGCACACCGAGCACGAGT |
| Havcr2 | AGTGGGAGTCTCTGCTGGGT |
|  | CATTTGCCAACCCTCCTGGA |
| Ptrf | GCCGGCCAGATAAAGAAACTG |
|  | CTCAGTTTGGCCGGCAGCTT |
| A2m | AGCCGGACGGAAGTCAGCAA |
|  | CTGGCTTCAGGTCTCTCACT |
| Igf1 | CGTCCCTATCGACAAACAAG |
|  | CCTCCTACATTCTGTAGGTC |
| Wt1 | TACTGACAGTTGCACAGGCA |
|  | GGTAGCTCCTAGGTTCATCT |
| Hsd11b1 | TCTTCCATGACGACATCCAC |
|  | GAGTAGGGAGCAATCATAGG |
| Beta-actin | GCTGTATTCCCCTCCATCGTG |
|  | CACGGTTGGCCTTAGGGTTCAG |
