# Supplementary material for: Transcriptomic Analysis Brings New Insight into the Biological Role of the Prion Protein during Mouse Embryogenesis
Source: PLoS One. 2011 Aug 15;6(8):e23253. doi: 10.1371/journal.pone.0023253 (PMC3156130; doi:10.1371/journal.pone.0023253)
Supplement: Table S2 — List of differentially expressed genes in Prnp -knockout embryos. Up-regulated genes are in green. Down-regulated genes are in red. (DOCX) [file pone.0023253.s004.docx]

| **Name** | **Gene_ID** | **E7.5 (fold change)** | **E6.5 (flod Change)** | **Name** | **Gene_ID** | **E7.5 (fold change)** | **E6.5 (flod Change)** |
| --- | --- | --- | --- | --- | --- | --- | --- |
| 170003OG11 | gi\|94367656\|ref\|XM_130735.7\| |  | 0,088235294 | LOC674994 | gi\|94364096\|ref\|XM_982827.1\| |  | 0,135501355 |
| 4933425M15 | gi\|31342302\|ref\|NM_175674.2\| | 6,025423729 | 0,061810155 | LOC676933 | gi\|149251186\|ref\|XR_031924.1\| |  | 28 |
| 5330417C22 | gi\|85701809\|ref\|NM_001033304.1\| |  | 6 | LOC677333 | gi\|149268680\|ref\|XR_034279.1\| |  | 0,201141853 |
| A2m | gi\|31342256\|ref\|NM_175628.2\| | 0,107888411 |  | LOC677333 | gi\|149269299\|ref\|XR_031734.1\| | 2,452018122 | 0,18236715 |
| AA474408 | gi\|149257659\|ref\|XR_035373.1\| |  | 0,210857332 | LOC677333 | gi\|149269258\|ref\|XR_031461.1\| |  | 0,201141851 |
| AAA74408 | gi\|149258180\|ref\|XR_035227.1\| |  | 0,173926475 | Lsamp | gi\|118130292\|ref\|NM_175548.3\| | 0,05625 |  |
| AAA74408 | gi\|149257938\|ref\|XR_035336.1\| |  | 0,19217196 | Lum | gi\|6678739\|ref\|NM_008524.1\| | 4,59375 |  |
| Abat | gi\|37202120\|ref\|NM_172961.2\| | 0,139175258 |  | Ly6A | gi\|31981636\|ref\|NM_010738.2\| | 0,09081987 |  |
| Abcd1 | gi\|6671496\|ref\|NM_007435.1\| | 0,13359375 |  | Ly-6c | gi\|149266926\|ref\|XM_001475001.1\| | 0,088857546 |  |
| Abp1 | gi\|22095012\|ref\|NM_029638.1\| | 0,325301205 |  | Ly6c2 | gi\|149266692\|ref\|XM_001477071.1\| | 0,088195077 |  |
| Acss1 | gi\|18034772\|ref\|NM_080575.1\| | 0,104381443 |  | Ly6c2 | gi\|149266694\|ref\|XM_001477088.1\| | 0,093171806 |  |
| Acvr1c | gi\|141802377\|ref\|NM_001033369.2\| | 3,401162791 | 0,18128655 | Ly6Cc1 | gi\|58331141\|ref\|NM_010741.2\| | 0,082687397 |  |
| Adam12 | gi\|117606340\|ref\|NM_007400.2\| | 0,16456117 |  | Ly6i | gi\|21356650\|ref\|NM_020498.1\| | 0,136014344 |  |
| Add3 | gi\|31542110\|ref\|NM_013758.2\| | 0,389918626 |  | Lyve1 | gi\|118131124\|ref\|NM_053247.4\| | 0,057177198 |  |
| Ado | gi\|53749203\|ref\|NM_001005419.1\| |  | 5,951219551 | Magel2 | gi\|94380522\|ref\|XM_622091.3\| |  | 0,331076 |
| Ahnak1 | gi\|61743960\|ref\|NM_009643.1\| | 0,426611566 |  | Mal | gi\|141801061\|ref\|NM_010762.4\| | 0,110119048 |  |
| Ahnak2 | gi\|85702000\|ref\|NM_001033476.1\| | 0,160714286 |  | Mesp1 | gi\|33469090\|ref\|NM_008588.1\| |  | 0,171122995 |
| Alox5 | gi\|116686109\|ref\|NM_009662.2\| | 0,099264706 |  | Mfap5 | gi\|118131047\|ref\|NM_015776.2\| | 0,127933846 |  |
| Amotl1 | gi\|124487106\|ref\|NM_001081395.1\| | 0,386870504 |  | Mgat4c | gi\|142385876\|ref\|NM_026243.3\| |  | 15,5 |
| Ampd3 | gi\|66792801\|ref\|NM_009667.2\| | 0,215324621 |  | Mgp | gi\|123701577\|ref\|NM_008597.3\| | 0,197949527 |  |
| Angpt2 | gi\|118131014\|ref\|NM_007426.3\| | 0,19054878 |  | Mib2 | gi\|110347530\|ref\|NM_145124.2\| | 5,011363636 | 0,067968185 |
| Angpt4 | gi\|6753005\|ref\|NM_009641.1\| | 0,096774194 |  | misc | gi\|94377878\|ref\|XR_005046.1\| | 0,088686131 |  |
| Ano1 | gi\|110835695\|ref\|NM_178642.4\| | 0,15173147 |  | Mixl1 | gi\|31543252\|ref\|NM_013729.2\| | 2,823033708 |  |
| Ano10 | gi\|141802585\|ref\|NM_133979.2\| | 0,348784195 |  | Mmp7 | gi\|144227218\|ref\|NM_010810.3\| | 7,78125 | 6,44012945 |
| Anxa1 | gi\|124517662\|ref\|NM_010730.2\| | 0,348123946 |  | Msln | gi\|9256567\|ref\|NM_018857.1\| | 0,016071429 |  |
| Anxa8 | gi\|142377277\|ref\|NM_013473.3\| | 0,07607582 |  | Mt3 | gi\|7305286\|ref\|NM_013603.1\| | 0,131971154 |  |
| Apob | gi\|110625670\|ref\|NM_009693.1\| | 2,318545316 |  | Mtap7d2 | gi\|124487456\|ref\|NM_001081124.1\| | 0,185096154 |  |
| Aqp1 | gi\|6680709\|ref\|NM_007472.1\| | 0,107876712 |  | Myoz1 | gi\|134032009\|ref\|NM_021508.3\| |  | 0,046605876 |
| Arl4a | gi\|87252728\|ref\|NM_001039515.1\| | 0,335106383 |  | Nampt | gi\|10946947\|ref\|NM_021524.1\| | 0,305389222 |  |
| Arl4a | gi\|87252732\|ref\|NM_007487.3\| | 0,302194149 |  | Napsa | gi\|6680551\|ref\|NM_008437.1\| | 26,4375 | 9,153153153 |
| Arrdc4 | gi\|112363103\|ref\|NM_001042592.2\| | 0,256542056 |  | Nipal1 | gi\|124487088\|ref\|NM_001081205.1\| | 0,176855895 |  |
| Atoh8 | gi\|118130066\|ref\|NM_153778.3\| | 0,212652439 |  | Nmnat2 | gi\|57863769\|ref\|NM_175460.3\| | 0,268442623 |  |
| Atp8b1 | gi\|95007005\|ref\|NM_001001488.3\| | 0,210638298 |  | Nrcam | gi\|142377989\|ref\|NM_176930.3\| | 0,240458015 |  |
| B3galnt1 | gi\|141803426\|ref\|NM_020026.3\| | 0,360768501 |  | Olfml3 | gi\|86439988\|ref\|NM_133859.2\| | 0,171821672 |  |
| BC015286 | gi\|142371927\|ref\|NM_198171.2\| |  | 6,9494949 | Parm1 | gi\|118130163\|ref\|NM_145562.2\| | 0,264209869 |  |
| Bche | gi\|124487349\|ref\|NM_009738.3\| | 0,154816514 |  | Parp3 | gi\|25014094\|ref\|NM_145619.2\| | 0,206701807 |  |
| Bcmo1 | gi\|11993945\|ref\|NM_021486.2\| | 0,327184814 |  | Pax8 | gi\|118130210\|ref\|NM_011040.3\| |  | 16,51851852 |
| Bdnf | gi\|114326458\|ref\|NM_001048142.1\| | 0,162 |  | Pcdh19 | gi\|94408163\|ref\|XM_975492.1\| |  | 0,0625 |
| Bmp2 | gi\|71896668\|ref\|NM_007553.2\| | 0,338832487 |  | Pcdh19 | gi\|149272245\|ref\|XM_001473606.1\| |  | 0,078431373 |
| C1qtnf1 | gi\|133892163\|ref\|NM_019959.2\| | 0,261627907 |  | Pcdh19 | gi\|149272244\|ref\|XM_205287.6\| |  | 0,0657277 |
| C2 | gi\|7304936\|ref\|NM_013484.1\| | 0,174418605 |  | Pdgfrl | gi\|42476141\|ref\|NM_026840.2\| | 0,194832402 |  |
| C3 | gi\|126518316\|ref\|NM_009778.2\| |  | 5,192214112 | Pdzk1ip1 | gi\|141802816\|ref\|NM_026018.2\| | 0,089502762 |  |
| C330006A16 | gi\|94366195\|ref\|XM_913936.2\| |  | 5,44973545 | Penk | gi\|141801808\|ref\|NM_001002927.2\| | 3,970588235 |  |
| C330006A16 | gi\|94366305\|ref\|XM_358556.5\| |  | 5,537777778 | Pfas | gi\|149261973\|ref\|XM_111232.8\| |  | 5,058013766 |
| Casp12 | gi\|142367382\|ref\|NM_009808.3\| | 0,177631579 |  | Pfas | gi\|149262172\|ref\|XM_908501.3\| |  | 5,37240971 |
| CD34 | gi\|19526791\|ref\|NM_133654.1\| | 0,148728814 |  | Pglyrp1 | gi\|118130401\|ref\|NM_009402.2\| | 0,094090909 |  |
| CD36 | gi\|142363407\|ref\|NM_007643.3\| | 0,243975904 |  | Pmaip1 | gi\|118130467\|ref\|NM_021451.2\| | 0,324928977 |  |
| CD55 | gi\|114326521\|ref\|NM_010016.2\| | 0,33801214 |  | Pou5f1 | gi\|125490391\|ref\|NM_013633.2\| | 2,554245283 |  |
| Cdh22 | gi\|31341388\|ref\|NM_174988.2\| |  | 0,045454545 | Ppp1r3c | gi\|33468956\|ref\|NM_016854.1\| | 0,055147059 |  |
| Cdx4 | gi\|141802151\|ref\|NM_007674.2\| | 0,252029221 |  | Prap1 | gi\|6678510\|ref\|NM_009475.1\| | 11,30769231 |  |
| Ceacam10 | gi\|142352593\|ref\|NM_007675.3\| | 0,153586957 |  | Prkg2 | gi\|142372298\|ref\|NM_008926.3\| | 0,127299331 |  |
| Chsy3 | gi\|124486748\|ref\|NM_001081328.1\| |  | 0,087719298 | Prl2a1 | gi\|9910513\|ref\|NM_019991.1\| |  | 0,128642309 |
| Cldn1 | gi\|142350296\|ref\|NM_016674.3\| | 0,062269373 |  | Prlpa | gi\|142364521\|ref\|NM_011165.2\| |  | 0,183750115 |
| Cldn10 | gi\|144226219\|ref\|NM_021386.3\| | 0,178217822 |  | Prps2 | gi\|141802916\|ref\|NM_026662.3\| | 0,269626556 |  |
| Cldn5 | gi\|142348145\|ref\|NM_013805.3\| | 0,152027027 |  | Prss28 | gi\|115299757\|ref\|NM_053259.2\| | 16,875 | 5,260273973 |
| Clu | gi\|7304966\|ref\|NM_013492.1\| |  | 6,038986355 | Prss29 | gi\|31981507\|ref\|NM_053260.2\| | 16,6875 | 9,117370892 |
| Col5a2 | gi\|86613789\|ref\|NM_007737.2\| | 0,182460612 |  | Psca | gi\|21312315\|ref\|NM_028216.1\| | 0,076737452 |  |
| Corin | gi\|142374600\|ref\|NM_016869.2\| | 0,05 |  | Ptges | gi\|46852157\|ref\|NM_022415.2\| | 0,117391304 |  |
| Crip1 | gi\|123701561\|ref\|NM_007763.3\| | 0,142356361 |  | Ptgs1 | gi\|144227245\|ref\|NM_008969.3\| | 0,106343284 |  |
| Cryaa | gi\|113931666\|ref\|NM_013501.2\| | 0,126168224 |  | Ptn | gi\|118130571\|ref\|NM_008973.2\| | 0,266915138 |  |
| Cryab | gi\|133892993\|ref\|NM_009964.2\| | 0,306270593 |  | Ptrf | gi\|71043973\|ref\|NM_008986.2\| | 0,120221546 | 13,72599784 |
| Cst9 | gi\|6753545\|ref\|NM_009979.1\| | 0 |  | Ramp3 | gi\|142384664\|ref\|NM_019511.2\| | 0,181985294 |  |
| Ctla2a | gi\|6681076\|ref\|NM_007796.1\| | 0,23944218 |  | Rax | gi\|89274178\|ref\|NM_013833.2\| |  | 0,022988506 |
| Ctla2b | gi\|149264174\|ref\|XM_001477566.1\| | 0,293968318 |  | Rgs2 | gi\|141802972\|ref\|NM_009061.3\| | 0,40685136 |  |
| Ctsk | gi\|142352209\|ref\|NM_007802.3\| | 0,389200899 |  | Rgs5 | gi\|141801698\|ref\|NM_009063.3\| | 0,310409699 |  |
| Ctso | gi\|118130454\|ref\|NM_177662.2\| | 0,181967213 |  | Rmrp | gi\|32526884\|ref\|NR_001460.1\| | 4,182368319 | 0,05879877 |
| Cubn | gi\|124487347\|ref\|NM_001081084.1\| | 2,290400552 |  | Rpph1 | gi\|46402165\|ref\|NR_002142.1\| | 3,08763587 | 0,114068441 |
| Cxcl14 | gi\|119392097\|ref\|NM_019568.2\| | 0,24535316 |  | Rufy3 | gi\|31542481\|ref\|NM_027530.2\| |  | 0,074504443 |
| Cyp11b1 | gi\|141803200\|ref\|NM_001033229.2\| | 0,135714286 |  | S100a4 | gi\|33859623\|ref\|NM_011311.1\| | 0,165395284 |  |
| Cyp2j11 | gi\|51921286\|ref\|NM_001004141.1\| | 0,05625 |  | Samd12 | gi\|144446081\|ref\|NM_177225.3\| | 0 |  |
| Cyp4v3 | gi\|118129905\|ref\|NM_133969.2\| | 0,105882353 |  | Samhd1 | gi\|46909601\|ref\|NM_018851.2\| | 0,326830443 |  |
| Dcn | gi\|142383942\|ref\|NM_007833.3\| | 0,113946241 |  | Sat1 | gi\|42476316\|ref\|NM_009121.3\| | 0,267679754 |  |
| Ddx58 | gi\|45598376\|ref\|NM_172689.2\| |  | 0,159221077 | Scn3b | gi\|144922642\|ref\|NM_178227.4\| |  | 0,072727273 |
| Degs2 | gi\|142345826\|ref\|NM_027299.3\| | 6,75 |  | Scrt2 | gi\|149250060\|ref\|XM_619828.4\| |  | 0,060606061 |
| Des | gi\|33563249\|ref\|NM_010043.1\| | 0,097111704 |  | SecTm1b | gi\|114145547\|ref\|NM_026907.3\| | 0 |  |
| Dhrs3 | gi\|142370009\|ref\|NM_011303.3\| | 0,377081888 |  | Serpina1e | gi\|76881810\|ref\|NM_009247.2\| | 32,625 |  |
| Dio3 | gi\|111494233\|ref\|NM_172119.2\| | 0,131875215 |  | Serping1 | gi\|142370111\|ref\|NM_009776.2\| | 0,258062561 |  |
| Dkk2 | gi\|66955883\|ref\|NM_020265.3\| | 0,24939759 |  | Sfrp4 | gi\|141803390\|ref\|NM_016687.2\| | 0,181071429 |  |
| Dmkn | gi\|142375786\|ref\|NM_172899.3\| | 0,099904853 |  | Sfrp5 | gi\|31560420\|ref\|NM_018780.2\| | 0,116330391 |  |
| Dmkn | gi\|50284531\|ref\|NM_028618.1\| | 0,098133287 |  | Sgk1 | gi\|6755489\|ref\|NM_011361.1\| | 0,286252711 |  |
| Dnajb5 | gi\|40254371\|ref\|NM_019874.3\| |  | 0,062222222 | Slc2a12 | gi\|142387928\|ref\|NM_178934.3\| | 0,187932028 |  |
| Dpep1 | gi\|6681216\|ref\|NM_007876.1\| | 0,123940678 |  | Slc6a12 | gi\|141802667\|ref\|NM_133661.2\| | 0,057175503 |  |
| Dscam | gi\|118130729\|ref\|NM_031174.3\| |  | 0,135658915 | Slco1a6 | gi\|12963796\|ref\|NM_023718.1\| | 11,475 |  |
| Dtna | gi\|95113657\|ref\|NM_207650.3\| | 0,245267624 |  | Slpi | gi\|141801540\|ref\|NM_011414.2\| | 0,02937788 |  |
| Ednrb | gi\|141802494\|ref\|NM_007904.3\| | 0,375659051 |  | Smoc2 | gi\|11612492\|ref\|NM_022315.1\| | 0,14516129 |  |
| Efemp1 | gi\|22122482\|ref\|NM_146015.1\| | 0,110915493 |  | Sned1 | gi\|40254324\|ref\|NM_172463.3\| | 0,299395161 |  |
| EG666182 | gi\|94363835\|ref\|XM_991070.1\| |  | 0,078431373 | Sorbs2 | gi\|124301205\|ref\|NM_172752.3\| | 0,177605969 |  |
| Ehd3 | gi\|141802831\|ref\|NM_020578.2\| | 0,231281726 |  | Sparcl1 | gi\|31982799\|ref\|NM_010097.2\| | 0,265478972 |  |
| Emcn | gi\|8393724\|ref\|NM_016885.1\| | 0,063508065 |  | Speer2 | gi\|144925922\|ref\|NM_173069.2\| |  | 0,009389671 |
| Eomes | gi\|83921571\|ref\|NM_010136.2\| | 2,338188559 |  | SqrdI | gi\|31981548\|ref\|NM_021507.4\| | 0,136732852 |  |
| Epdr1 | gi\|142372189\|ref\|NM_134065.3\| | 0,213235294 |  | Srd5a1 | gi\|87044894\|ref\|NM_175283.3\| | 0,21231203 |  |
| Fads3 | gi\|70887800\|ref\|NM_021890.3\| | 0,294098985 |  | Srgn | gi\|118129990\|ref\|NM_011157.2\| | 0,288227392 |  |
| Fam154b | gi\|134288849\|ref\|NM_177894.4\| |  | 0,076923077 | St8sia4 | gi\|6677968\|ref\|NM_009183.1\| | 0,183476395 |  |
| Fam186b | gi\|124486942\|ref\|NM_001081254.1\| |  | 0,021231423 | Stac3 | gi\|141803518\|ref\|NM_177707.3\| |  | 0,074829932 |
| Fbln2 | gi\|124517692\|ref\|NM_001081437.1\| | 0,143649194 |  | Suox | gi\|31343458\|ref\|NM_173733.2\| | 0,146511628 |  |
| Fbln2 | gi\|124517703\|ref\|NM_007992.2\| | 0,139992504 |  | Tacc1 | gi\|110681725\|ref\|NM_199323.2\| | 0,371178344 |  |
| Fbn1 | gi\|118197276\|ref\|NM_007993.2\| | 0,13909409 |  | Tacc1 | gi\|110681726\|ref\|NM_177089.4\| | 0,4375 |  |
| Fgf5 | gi\|141802811\|ref\|NM_010203.3\| | 4,203125 |  | Tdgf1 | gi\|134053944\|ref\|NM_011562.2\| | 3,779442149 |  |
| Fmo1 | gi\|31981773\|ref\|NM_010231.2\| | 0,311621529 |  | Tdo2 | gi\|31982696\|ref\|NM_019911.2\| | 0,097258917 |  |
| Fosl2 | gi\|40789304\|ref\|NM_008037.3\| | 0,268508287 |  | Tgfbr2 | gi\|27363473\|ref\|NM_009371.2\| | 0,162145091 |  |
| Fstl1 | gi\|141802756\|ref\|NM_008047.3\| | 0,289543229 |  | Them4 | gi\|110626049\|ref\|NM_029431.1\| | 0,323054475 |  |
| Gatm | gi\|142354911\|ref\|NM_025961.3\| | 0,108279252 |  | Thy1 | gi\|134152684\|ref\|NM_009382.3\| | 0,13959854 |  |
| Gcnt3 | gi\|124430728\|ref\|NM_028087.2\| | 0,197260274 |  | Timp3 | gi\|119637812\|ref\|NM_011595.2\| | 0,419726615 |  |
| Gda | gi\|87299644\|ref\|NM_010266.2\| | 0,13784585 |  | Tlr3 | gi\|144227205\|ref\|NM_126166.3\| | 0,160714286 |  |
| Ggtla1 | gi\|141801950\|ref\|NM_011820.3\| | 0,168449198 |  | Tlr4 | gi\|118130391\|ref\|NM_021297.2\| | 0,145334928 |  |
| Gimap4 | gi\|56549086\|ref\|NM_174990.3\| | 0,118421053 |  | Tm4sf1 | gi\|88900519\|ref\|NM_008536.3\| | 0,181208054 |  |
| Gja1 | gi\|21687178\|ref\|NM_010288.2\| | 0,327582663 |  | Tmem102 | gi\|142360242\|ref\|NM_001033433.2\| |  | 6,145833333 |
| Gm996 | gi\|53749207\|ref\|NM_001005424.1\| |  | 0,11954023 | Tmem154 | gi\|31343187\|ref\|NM_177260.2\| | 0,089760638 |  |
| Gpihbp1 | gi\|58037120\|ref\|NM_026730.1\| | 0,132221116 |  | Tmem176a | gi\|142353616\|ref\|NM_025326.3\| | 0,1575 |  |
| Gpnmb | gi\|142371734\|ref\|NM_053110.3\| | 0,325427683 |  | Tmem176b | gi\|142372371\|ref\|NM_023056.3\| | 0,149765074 |  |
| Gpr115 | gi\|149269311\|ref\|XM_001476141.1\| | 0,070081967 |  | Tmem204 | gi\|47564089\|ref\|NM_001001183.1\| | 0,12347561 |  |
| Gpr115 | gi\|149268852\|ref\|XM_894986.3\| | 0,093418728 |  | Tnfaip2 | gi\|6678374\|ref\|NM_009396.1\| | 0,330967742 |  |
| Gpr64 | gi\|119943130\|ref\|NM_001079857.1\| | 0,092465753 |  | Tnfaip3 | gi\|31543879\|ref\|NM_009397.2\| | 0,029605263 |  |
| Gpx3 | gi\|6680076\|ref\|NM_008161.1\| | 0,179568168 |  | Tnfrsf11b | gi\|113930715\|ref\|NM_008764.3\| | 0,092022845 |  |
| Gsdmds1 | gi\|142348546\|ref\|NM_026960.2\| | 0,185126582 |  | Trim16 | gi\|70778827\|ref\|NM_053169.2\| | 0,201315789 |  |
| H2-M10.1 | gi\|34328212\|ref\|NM_013544.2\| |  | 0,024691358 | Tshz2 | gi\|61097921\|ref\|NM_080455.1\| | 0,271302251 |  |
| Havcr2 | gi\|21703359\|ref\|NM_134250.1\| | 0,052380952 |  | Tspan2 | gi\|70608156\|ref\|NM_027533.2\| | 0,211538462 |  |
| Hba-x | gi\|142350815\|ref\|NM_010405.3\| | 0,178977273 |  | Ugt1a1 | gi\|47059134\|ref\|NM_201645.1\| | 0,061363636 |  |
| Hdac9 | gi\|70778860\|ref\|NM_024124.2\| | 0,14673913 |  | Ugt1a6b | gi\|47059132\|ref\|NM_201410.1\| | 0,084375 |  |
| Hdc | gi\|142385676\|ref\|NM_008230.5\| |  | 8 | Ugt1a7c | gi\|141803423\|ref\|NM_201642.3\| | 0,102272727 |  |
| Hist1h2ao | gi\|149263930\|ref\|XM_978341.2\| | 2,895220588 |  | Usp53 | gi\|142371445\|ref\|NM_133857.2\| | 0,386485927 |  |
| Hist1h4h | gi\|141802886\|ref\|NM_153173.2\| |  | 0,124610592 | Vldlr | gi\|7305628\|ref\|NM_013703.1\| | 0,214506173 |  |
| Hist2h2bb | gi\|68226432\|ref\|NM_175666.2\| |  | 0,104938272 | Vsig2 | gi\|114145510\|ref\|NM_020518.2\| | 0,141428571 |  |
| Hist2h3b | gi\|30061346\|ref\|NM_178215.1\| |  | 0,146666667 | Wt1 | gi\|120444921\|ref\|NM_144783.2\| | 0,280164093 |  |
| Hist2h3c1 | gi\|30061400\|ref\|NM_178216.1\| |  | 0,131067961 | Zbtb20 | gi\|9790132\|ref\|NM_019778.1\| | 0,259322034 |  |
| Hist2h3c2-ps | gi\|34328342\|ref\|NM_054045.2\| |  | 0,128266033 | Zfp109 | gi\|9931981\|ref\|NM_020262.1\| |  | 0,126394052 |
| Hist2h4 | gi\|21361208\|ref\|NM_033596.1\| |  | 0,119298246 |  | gi\|149256881\|ref\|XM_001478066.1\| | 0,083333333 |  |
| Hist4h4 | gi\|28316745\|ref\|NM_175652.1\| |  | 0,084656085 |  | gi\|94374443\|ref\|XM_909130.2\| | 0,271011777 |  |
| Hmx2 | gi\|110625930\|ref\|NM_145998.2\| |  | 0,094623656 |  | gi\|149266696\|ref\|XM_001477102.1\| | 0,100067385 |  |
| Hoxa10 | gi\|6680242\|ref\|NM_008263.1\| | 0,200342466 |  |  | gi\|142376574\|ref\|NM_177599.3\| | 0,010613208 |  |
| Hoxa11 | gi\|118129999\|ref\|NM_010450.2\| | 0,262019231 |  |  | gi\|149263928\|ref\|XM_001475956.1\| | 0,283308931 |  |
| Hoxd10 | gi\|141802469\|ref\|NM_013554.3\| | 0,284520349 |  |  | gi\|118129953\|ref\|NM_172777.2\| | 0,097355769 |  |
| Hoxd8 | gi\|112807183\|ref\|NM_008276.2\| | 0,1375 |  |  | gi\|110626043\|ref\|NM_029142.1\| | 0,115935115 |  |
| Hsd11b1 | gi\|113680740\|ref\|NM_008288.2\| | 0,056654676 |  |  | gi\|149260864\|ref\|XM_001478148.1\| | 0,251493174 |  |
| Hsd11b1 | gi\|113680660\|ref\|NM_001044751.1\| | 0,055509868 |  |  | gi\|126506315\|ref\|NM_001081963.1\| | 0,324369662 |  |
| Hspb7 | gi\|142351368\|ref\|NM_013868.3\| | 0,016071429 |  |  | gi\|149268975\|ref\|XR_033381.1\| | 0,274172185 |  |
| Htra3 | gi\|110815868\|ref\|NM_0320127.\| | 0,122113164 |  |  | gi\|149251850\|ref\|XR_034037.1\| | 0,075721154 |  |
| Igf1 | gi\|133892662\|ref\|NM_010512.3\| | 0,201988636 |  |  | gi\|149263684\|ref\|XM_001477593.1\| | 0,197519084 |  |
| Igfbp7 | gi\|62990156\|ref\|NM_008048.2\| | 0,253863988 |  |  | gi\|94399046\|ref\|XM_909906.2\| | 0,124615385 |  |
| Igsf11 | gi\|40254204\|ref\|NM_170599.2\| | 0,275943396 |  |  | gi\|149272419\|ref\|XM_001472704.1\| | 0,280290102 |  |
| Il6ra | gi\|110431355\|ref\|NM_010559.2\| | 0,172830725 |  |  | gi\|94374423\|ref\|XM_908115.2\| | 0,261178862 |  |
| Indol1 | gi\|22122378\|ref\|NM_145949.1\| |  | 0,059139785 |  | gi\|110625808\|ref\|NM_027627.1\| | 0,128076923 |  |
| Irak3 | gi\|142380077\|ref\|NM_028679.3\| | 0,260714286 |  |  | gi\|94374638\|ref\|XM_485586.4\| | 0,249092559 |  |
| Jam2 | gi\|142352774\|ref\|NM_023844.3\| | 0,192307692 |  |  | gi\|142351874\|ref\|NM_145450.3\| | 0,231617647 |  |
| Kazald1 | gi\|142353193\|ref\|NM_178929.3\| | 0,179560261 |  |  | gi\|149265290\|ref\|XM_910675.3\| | 0,0859375 |  |
| Kcnc1 | gi\|76677931\|ref\|NM_008421.2\| |  | 0,104868914 |  |  |  |  |
| Kcnd3 | gi\|86991433\|ref\|NM_001039347.1\| | 0,029220779 |  |  | gi\|149264700\|ref\|XR_032778.1\| | 0,036684783 |  |
| Klk14 | gi\|33469048\|ref\|NM_174866.1\| | 0,075 |  |  | gi\|149269704\|ref\|XM_001476237.1\| | 0,225308642 |  |
| Krt84 | gi\|33563237\|ref\|NM_008474.1\| | 0,16487069 |  |  | gi\|94400800\|ref\|XM_918770.2\| | 0,116219008 |  |
| Lamb2 | gi\|31982222\|ref\|NM_008483.2\| | 0,328125 |  |  | gi\|94400799\|ref\|XM_904281.2\| | 0,117356115 |  |
| Lbp | gi\|113865990\|ref\|NM_008489.2\| | 0,092574009 |  |  | gi\|31342510\|ref\|NM_178098.2\| | 0,188752087 |  |
| Ldhd | gi\|34328378\|ref\|NM_027570.3\| | 0,113924051 |  |  | gi\|94400798\|ref\|XM_918758.2\| | 0,10926259 |  |
| Lgr4 | gi\|124430558\|ref\|NM_172671.2\| | 0,406495468 |  |  | gi\|142360631\|ref\|NM_178737.4\| | 2,808566434 |  |
| Lims2 | gi\|142347134\|ref\|NM_144862.2\| | 0,078488372 |  |  | gi\|149249534\|ref\|XR_034465.1\| | 0,292682927 |  |
| LOC100043825 | gi\|149265870\|ref\|XM_001481034.1\| |  | 0,055345912 |  | gi\|149256063\|ref\|XM_0389.101479\| | 0,293478261 |  |
| LOC100046008 | gi\|149250380\|ref\|XM_001475350.1\| |  | 0,068728522 |  | gi\|149261778\|ref\|XM_001475752.1\| | 0,199675325 |  |
| LOC100046045 | gi\|149270001\|ref\|XR_032266.1\| |  | 0,04 |  | gi\|149264283\|ref\|XM_001477652.1\| | 0,257890365 |  |
| LOC100046120 | gi\|149266011\|ref\|XM_001475611.1\| |  | 9,84 |  | gi\|142363148\|ref\|NM_027519.2\| | 0,354697452 |  |
| LOC100047285 | gi\|149269087\|ref\|XM_001477507.1\| | 9 | 9,477477477 |  | gi\|149250374\|ref\|XM_001475472.1\| | 0,384427374 |  |
| LOC100047300 | gi\|149260446\|ref\|XM_001478776.1\| | 3,476694915 |  |  | gi\|142360223\|ref\|NM_024283.2\| | 12,65625 |  |
| LOC100048759 | gi\|149274892\|ref\|XR_035041.1\| |  | 6,302325581 |  | gi\|149256635\|ref\|XM_001480672.1\| | 0,336267606 |  |
| LOC383326 | gi\|149269761\|ref\|XR_033450.1\| |  | 5,490636704 |  | gi\|149266741\|ref\|XM_001478533.1\| | 0 |  |
| LOC435145 | gi\|94398806\|ref\|XM_487043.4\| |  | 0,091503268 |  | gi\|149251752\|ref\|XM_001471642.1\| | 0,139655172 |  |
| LOC435145 | gi\|94399138\|ref\|XM_987106.1\| |  | 0,111111111 |  | gi\|149269644\|ref\|XM_001474216.1\| | 0,293778802 |  |
| LOC627701 | gi\|94372774\|ref\|XM_892400.2\| |  | 0,168955472 |  | gi\|149269617\|ref\|XM_001473270.1\| | 0,3366 |  |
| LOC633979 | gi\|149260699\|ref\|XR_032514.1\| |  | 0,028571429 |  | gi\|149251615\|ref\|XM_001471696.1\| | 0,180944056 |  |
| LOC633979 | gi\|149260818\|ref\|XR_035504.1\| |  | 0,080808081 |  | gi\|149260757\|ref\|XM_001478007.1\| | 0,26 |  |
| LOC636319 | gi\|149270024\|ref\|XR_032953.1\| |  | 5,56097561 |  | gi\|149261822\|ref\|XM_001472216.1\| | 0,115056818 |  |
| LOC666964 | gi\|94395360\|ref\|XM_987269.1\| |  | 0,119298246 |  | gi\|149263982\|ref\|XM_904319.2\| | 0,372335025 |  |
|  |  |  |  |  | gi\|149233590\|ref\|XM_001472850.1\| | 0,421314741 |  |
|  |  |  |  |  | gi\|21311852\|ref\|NM_028747.1\| | 0,199367089 |  |
|  |  |  |  |  |  |  |  |
|  |  | | | | | | |
|  |  | |  |  |  |  |  |
|  |  | |  |  |  |  |  |
|  |  |  |  |  |  |  |  |
|  |  |  |  |  |  |  |  |
|  |  |  |  |  |  |  |  |
|  |  |  |  |  |  |  |  |
|  |  |  |  |  |  |  |  |
|  |  |  |  |  |  |  |  |
